# Synchronous and metachronous multiple primary cancers in melanoma survivors: a gender perspective

**DOI:** 10.3389/fpubh.2023.1195458

**Published:** 2023-06-16

**Authors:** Stefano Guzzinati, Alessandra Buja, Giulia Grotto, Manuel Zorzi, Mariagiovanna Manfredi, Eleonora Bovo, Paolo Del Fiore, Saveria Tropea, Luigi Dall’Olmo, Carlo R. Rossi, Simone Mocellin, Massimo Rugge

**Affiliations:** ^1^Veneto Tumor Registry, Azienda Zero, Padua, Italy; ^2^Department of Cardiac, Thoracic and Vascular Sciences, and Public Health, University of Padua, Padua, Italy; ^3^Soft-Tissue, Peritoneum, and Melanoma Surgical Oncology Unit, Veneto Institute of Oncology IOV-IRCCS, Padua, Italy; ^4^Department of Surgery, Oncology and Gastroenterology - DISCOG, University of Padua, Padua, Italy; ^5^Department of Medicine - DIMED, Pathology and Cytopathology Unit, University of Padua, Padua, Italy

**Keywords:** cutaneous melanoma, multiple cancers, second primary cancer, subsequent primary cancer, metachronous cancer, synchronous cancer

## Abstract

**Background:**

Long-term survivors of cutaneous malignant melanoma (CMM) risk subsequent malignancies due to both host-related and environmental risk factors. This retrospective population-based study differentially assesses the risk of synchronous and metachronous cancers in a cohort of CMM survivors stratified by sex.

**Methods:**

The cohort study (1999–2018) included 9,726 CMM survivors (M = 4,873, F = 4,853) recorded by the cancer registry of all 5,000,000 residents in the Italian Veneto Region. By excluding subsequent CMM and non-CMM skin cancers, the incidence of synchronous and metachronous malignancies was calculated according to sex and tumor site, standardizing for age and calendar year. The Standardized Incidence Ratio (SIR) was calculated as the ratio between the number of subsequent cancers among CMM survivors and the expected number of malignancies among the regional population.

**Results:**

Irrespective of the site, the SIR for synchronous cancers increased in both sexes (SIR = 1.90 in males and 1.73 in females). Both sexes also demonstrated an excess risk for synchronous kidney/urinary tract malignancies (SIR = 6.99 in males and 12.11 in females), and women had an increased risk of synchronous breast cancer (SIR = 1.69). CMM male survivors featured a higher risk of metachronous thyroid (SIR = 3.51, 95% CI [1.87, 6.01]), and prostate (SIR = 1.35, 95% CI [1.12, 1.61]) malignancies. Among females, metachronous cancers featured higher SIR values than expected: kidney/urinary tract (SIR = 2.27, 95% CI [1.29, 3.68]), non-Hodgkin’s lymphoma (SIR = 2.06, 95% CI [1.24, 3.21]), and breast (SIR = 1.46, 95% CI [1.22, 1.74]). Females had an overall increased risk of metachronous cancers in the first 5 years after CMM diagnosis (SIR = 1.54 at 6–11 months and 1.37 at 1–5 years).

**Conclusion:**

Among CMM survivors, the risk of metachronous non-skin cancers is higher than in the general population and differs significantly by sex. These results encourage sex-tailored interventions for metachronous secondary cancer prevention.

## Introduction

Over the last few decades, we have seen a constant increase in cutaneous malignant melanoma (CMM), particularly among fair-skinned populations (1). Accounting for 5.6% of all newly-diagnosed cancer cases, CMM is currently the fifth most common type of cancer worldwide (2).

In the United States alone, the incidence of CMM went from 7.9/100,000 in 1975 to 25.3/100,000 in 2018, more than a 320% increase. In Italy in 2020, CMM was the third most common malignancy, with 14,900 new cases and a 5 year survival rate of 88% for men and 91% for women (3). In the same year, 169,900 people were living with a positive CMM diagnosis (3).

In CMM, as with other cancers, long-term survivors risk subsequent malignancies due to both host-related and environmental risk factors (4–6). A previous meta-analysis addressing the risk of developing subsequent cancers in CMM survivors showed an overall increased cancer risk of 1.57 (95% CI [1.29, 1.90]) (5). The most involved secondary primary sites were as follows: soft tissue, non-melanoma skin cancer, bone, non-Hodgkin’s lymphoma, kidney, prostate, female breast and colon-rectum (5).

Although the increased risk of developing second primary cancers in CMM survivors is known, we still need to analyze the different temporal onset patterns of subsequent malignancies, including a gender-based point of view. Also, studies on multiple primary cancers may provide powerful insights into cancer etiology, including the cancer-promoting role of environmental and genetic risk factors, and may offer the clinical rationale for developing secondary prevention strategies (including counseling), as well as decision-supporting tools. The current retrospective population-based study differentially assesses the risk of synchronous and metachronous cancers in a cohort of CMM survivors stratified by sex.

## Methods

### Study population

In Northeast Italy, the Veneto region covers approximately 18,345 square kilometers, with a resident population of over 4.8 million (7). Mortality, measured by the standardized death rate, is lower than the national average (7.9 vs. 8.2 per 1,000 inhabitants in 2016) (8) with the main causes of death represented by cancer and cardiovascular disease (9). The Regional healthcare system is based on the fundamental values of universality, free access, freedom of choice, pluralism of supply and equity (9).

### Data sources

Data for the study were retrieved from the population-based dataset in the Veneto Cancer Registry (“*Registro Tumori Veneto*” [RTV]), which was first established in 1987. The population coverage increased from 1,154,000 inhabitants in 1987 to nearly five million (encompassing the entire regional population) in 2014 (see Supplementary Table S1). The fraction of the population covered by the Registry has risen from 53% in 2008–2013 to 100% (approximately 5 million residents) from 2014 onwards. Supplementary Table S2 shows that the older registration area gave cancer incidence estimates that were comparable with the more modern data available for the whole population.

This retrospective cohort study included all CMM patients diagnosed from January 1st, 1999, to December 31st, 2018. All multiple (synchronous and metachronous) primary malignancies except for skin cancers (melanomatous and non-melanomatous) were considered. An interval time of 6 months from the index-CMM was used to distinguish synchronous (time elapsed ≤6 months) versus metachronous (time elapsed >6 months) multiple malignancies (6, 10, 11).

### Statistics

Cancer incidence rates were calculated from the RTV database, stratifying patients by sex, malignancy site, age bracket (0–4, 5–9, 10–14, … 85+ years) and calendar year group (1999–2003, 2004–2008, 2009–2013, 2014–2018). The Standardized Incidence Ratio (SIR) was calculated as the ratio between the observed cancers and the number that would be expected based on the incidence rates for the general population (12). The observed number of cases was defined as the number of all malignancies (excluding melanoma and non-melanoma skin cancers) diagnosed in the cohort. The occurrence of three or more cancers was not investigated, due to its negligible incidence (less than 1% of the general population).

To calculate the expected number of malignancies, the accumulated person-years at risk (PY) was multiplied by the rates to be expected if CMM survivors experienced the same cancer rates as the general reference population. The PY were defined as the period between a patient’s melanoma diagnosis and one of these following events: a second cancer diagnosis, death, or the end of the period considered (i.e., December 31st, 2018). Byar’s accurate approximation to the exact Poisson distribution was used to calculate 95% confidence intervals (95% CI) (13). The Benjamini and Hochberg False Discovery Rate correction was adopted to adjust for multiple comparisons (14). To assess the true excess of burden of second cancers in the population of CMM survivors, the Absolute Excess Risk (AER) per 1,000 PY was also obtained using the formula:

[(observed number – expected number) ÷ PY] × 1,000.

Results were deemed statistically significant at the *p* < 0.05 level. The Multiple Primary – Standardized Incidence Ratios session of SEER*Stat 8.4.0 (a publicly-available, interactive, Windows-based program produced by NCI) was used for the analysis (15).

## Results

Between January 1st, 1999 and December 31st, 2018, 9,726 CMM patients (M = 4,873, *F* = 4,853) were retrospectively considered. In 65,046 PY, 833 s malignancies (92 synchronous and 741 metachronous) were recorded.

Both synchronous (M:F = 61:31, *p* = 0.0018) and metachronous (M:F = 412:329, *p* = 0.0018) malignancies were more common among males.

The overall Standardized Incidence Ratio (SIR) was 1.84 (95% CI [1.48, 2.26]) for synchronous and 1.12 (95% CI [1.04, 1.21]) for metachronous malignancies. The Absolute Excess Risk (AER) per 1,000 PY was 8.93 (95% CI [4.00, 13.85]) for synchronous and 1.33 (95% CI [0.12, 2.54]) for metachronous cancers.

### Synchronous cancers

Irrespective of the cancer site, the SIR of synchronous cancers was higher than expected in males (SIR = 1.90, 95% CI [1.45, 2.44]) and females (SIR = 1.73, 95% CI [1.17, 2.45]) (Table 1).

**Table 1:** SIR of second cancers at different time points after a diagnosis of melanoma (significant estimates in bold).

Sex	Second malignancy site	Follow-up time since diagnosis months		Follow-up time interval since diagnosis months				
		Overall (synchronous and metachronous)	Overall (metachronous)	Synchronous	Metachronous			
		≥0	≥7	0–6	7–12	13–60	61–120	120+
Males	Total	1.09	1.03	**1.90**	1.29	1.01	1.05	0.92
	Prostate	**1.37**	**1.35**	1.55	1.69	**1.36**	1.32	1.24
	Kidney/urinary tract	**1.68**	1.26	**6.99**	3.05	1.53	0.52	1.19
	Thyroid	**3.77**	**3.51**	7.29	**11.75**	**3.16**	3.38	1.46
Females	Total	**1.30**	**1.27**	**1.73**	**1.54**	**1.37**	1.15	1.17
	Breast	**1.48**	**1.46**	**1.69**	1.26	**1.54**	1.34	**1.55**
	Kidney/urinary tract	**2.91**	**2.27**	**12.11**	**6.47**	2.10	2.31	1.28
	NHL	**2.12**	**2.06**	3.10	3.31	1.33	2.47	2.44

Skin melanomatous and non-melanomatous malignancies are excluded. NHL, non-Hodgkin’s lymphoma.

Both sexes featured an increased risk, compared to the general reference population, of synchronous kidney/urinary tract cancer (SIR in males = 6.99, 95% CI [3.35, 12.86]; SIR in females = 12.11, 95% CI [4.42, 26.36]). Females also had an increased risk of synchronous breast cancer.

### Metachronous cancers in males

Between the 7th month and the end of the 12th month, the SIR of metachronous cancers was 1.29 (95% [CI 0.92, 1.78]), while from the 13th to the 60th month from the CMM index, and the SIR was 1.01 (95% CI [0.86, 1.17]).

For metachronous cancer sites, the SIRs for prostate (1.35, 95% CI [1.12, 1.61]) and thyroid (3.51, 95% CI [1.87, 6.01]) cancers were significantly higher than expected (Table 2). Consistent results were obtained when the Absolute Excess of Risk (AER) per 1,000 PY was assessed (prostate: 1.10, 95% CI [0.11, 2.10]; thyroid: 0.33, 95% CI [0.05, 0.61]).

**Table 2:** SIR and AER per 1,000 PYs of second metachronous cancers after a diagnosis of melanoma (significant estimates in bold).

Second malignancy site	Males					Females				
	O	SIR	95% CI	AER per 1,000 PY	95% CI	O	SIR	95% CI	AER per 1,000 PY	95% CI
Total	412	1.03	(0.93, 1.13)	0.37	(−1.58, 2.32)	329	**1.27**	**(1.14, 1.41)**	**2.19**	**(0.71, 3.67)**
Oral cavity	8	0.71	(0.31, 1.40)	−0.11	(−0.41, 0.19)	5	1.23	(0.40, 2.88)	0.03	(−0.16, 0.21)
Esophagus	5	0.85	(0.28, 1.99)	−0.03	(−0.26, 0.20)	1	0.65	(0.01, 3.64)	−0.02	(−0.11, 0.08)
Stomach	12	0.83	(0.43, 1.46)	−0.08	(−0.44, 0.27)	6	0.83	(0.30, 1.8)	−0.04	(−0.26, 0.18)
Colon	38	1.00	(0.71, 1.38)	0.00	(−0.59, 0.60)	27	1.15	(0.75, 1.67)	0.11	(−0.33, 0.54)
Rectum	8	0.55	(0.24, 1.08)	−0.23	(−0.56, 0.10)	11	1.41	(0.70, 2.52)	0.10	(−0.17, 0.37)
Liver	12	0.63	(0.32, 1.10)	−0.25	(−0.63, 0.14)	4	0.71	(0.19, 1.81)	−0.05	(−0.24, 0.14)
Pancreas	10	0.69	(0.33, 1.27)	−0.16	(−0.50, 0.18)	14	1.28	(0.70, 2.14)	0.10	(−0.21, 0.40)
Lung	51	0.87	(0.65, 1.14)	−0.27	(−0.99, 0.45)	22	1.20	(0.75, 1.82)	0.12	(−0.27, 0.51)
Bone	1	2.13	(0.03, 11.84)	0.02	(−0.06, 0.10)	1	3.03	(0.04, 16.86)	0.02	(−0.05, 0.09)
Mesotheliomas	2	0.87	(0.10, 3.14)	−0.01	(−0.15, 0.13)	1	1.72	(0.02, 9.59)	0.01	(−0.06, 0.09)
Soft tissue	4	1.75	(0.47, 4.49)	0.06	(−0.11, 0.23)	4	3.03	(0.82, 7.76)	0.08	(−0.06, 0.23)
Breast	1	0.98	(0.01, 5.45)	0.00	(−0.10, 0.10)	126	**1.46**	**(1.22, 1.74)**	**1.25**	**(0.36, 2.15)**
Prostate	**121**	**1.35**	**(1.12, 1.61)**	**1.10**	**(0.11, 2.10)**	–				
Testis	2	1.47	(0.17, 5.31)	0.02	(−0.10, 0.15)	–				
Other male genitalia	1	5.88	(0.08, 32.73)	0.03	(−0.05, 0.10)	–				
Cervix uteri	–					4	1.42	(0.38, 3.64)	0.04	(−0.12, 0.20)
Corpus uteri	–					12	1.03	(0.53, 1.81)	0.01	(−0.29, 0.31)
Ovary	–					8	1.10	(0.47, 2.17)	0.02	(−0.22, 0.26)
Kidney/urinary tract	23	1.26	(0.80, 1.89)	0.17	(−0.27, 0.61)	16	**2.27**	**(1.29, 3.68)**	0.28	(−0.01, 0.58)
Urinary Bladder	42	1.02	(0.74, 1.39)	0.04	(−0.59, 0.66)	11	1.36	(0.68, 2.43)	0.09	(−0.18, 0.36)
Eye	3	4.69	(0.94, 13.7)	0.08	(−0.05, 0.21)	1	2.44	(0.03, 13.57)	0.02	(−0.05, 0.09)
Brain and other CNS sites	3	0.49	(0.10, 1.44)	−0.11	(−0.32, 0.10)	5	1.30	(0.42, 3.02)	0.04	(−0.15, 0.22)
Thyroid	**13**	**3.51**	**(1.87, 6.01)**	**0.33**	**(0.05, 0.61)**	14	1.40	(0.76, 2.34)	0.12	(−0.18, 0.43)
Hodgkin’s lymphoma	3	2.42	(0.49, 7.07)	0.06	(−0.08, 0.20)	2	2.06	(0.23, 7.44)	0.03	(−0.07, 0.14)
Non-Hodgkin’s lymphoma (NHL)	14	1.05	(0.57, 1.76)	0.02	(−0.34, 0.38)	19	**2.06**	**(1.24, 3.21)**	0.31	(−0.02, 0.63)
Multiple myeloma	2	0.36	(0.04, 1.31)	−0.12	(−0.31, 0.07)	3	0.78	(0.16, 2.28)	−0.03	(−0.19, 0.13)
Leukemia	8	1.01	(0.43, 1.99)	0.00	(−0.27, 0.28)	5	1.00	(0.32, 2.34)	0.00	(−0.19, 0.19)
Myeloproliferative diseases	4	1.36	(0.37, 3.48)	0.04	(−0.14, 0.22)	3	1.27	(0.25, 3.70)	0.02	(−0.12, 0.16)
Myelodysplastic syndromes	2	0.60	(0.07, 2.18)	−0.05	(−0.20, 0.11)	2	1.18	(0.13, 4.25)	0.01	(−0.11, 0.13)

Skin melanomatous and non-melanomatous malignancies are excluded. O: observed cancers.

The SIR of metachronous prostate cancer was higher in the 13–60 month interval (SIR = 1.36, 95% CI [1.02, 1.78]). Similar findings were also obtained for thyroid malignancies (SIR in the interval time of 7–12 months = 11.75, 95% CI [2.36, 34.37]; SIR in the interval time of 1–5 years = 3.16, 95% CI [1.02, 7.38]).

### Metachronous cancers in females

Regardless of the cancer site, both SIR (1.27, 95% CI [1.14, 1.41]) and AER per 1,000 PY (2.19, 95% CI [0.71, 3.67]) showed an increased risk of metachronous malignancies. Between the 7th and the end of the 12th month and from the 13th to the 60th month, the SIR for metachronous cancers were 1.54 (95% CI [1.01, 1.44]) and 1.37 (95% CI [1.16, 1.62]), respectively (Table 1).

When considering individual cancer sites, breast (SIR: 1.46, 95% CI [1.22, 1.74]) kidney/urinary tract (SIR: 2.27, 95% CI [1.29, 3.68]), and hematological (i.e., non-Hodgkin’s lymphoma [NHL] SIR: 2.06, 95% CI [1.24, 3.21]) featured significantly higher SIRs. The AER of metachronous breast cancers was 1.25 (95% CI [0.36, 2.15]), while the AER per 1,000 PY for kidney/urinary tract (0.28, 95% CI [0.01, 0.58]) and for NHL (0.31, 95% CI [0.02, 0.63]) were not significant.

The SIRs of metachronous breast cancer were found to be significantly higher in the 13–60 month interval (1.54, 95% CI [1.16, 2.01]) as well as more than 10 years after diagnosis (1.55, 95% CI [1.04, 2.23]). Similarly, the SIRs of metachronous kidney/urinary malignancies were also significant at 7–12 months from diagnosis (6.47, 95% CI [1.30, 18.89]).

## Discussion

This population-based cohort study analyzed data for 9,726 CMM survivors and found that the risk for synchronous cancers increased in both sexes, irrespective of cancer site. An excess risk for synchronous kidney/urinary tract malignancies was detected in both sexes, while women also had an increased risk of synchronous breast cancer. Concerning metachronous cancers, male survivors had a higher risk of thyroid and prostate malignancies, while females had an increased risk of kidney/urinary tract cancer, non-Hodgkin’s lymphoma, and breast cancer. Females had also an overall increased risk of metachronous cancers in the first 5 years after CMM diagnosis.

In western populations, the rising incidence of cancer and the increasing number of cancer survivors (which are frequently exposed to adjuvant carcinogenic therapies) has in turn increased the number of multiple primary malignancies (6, 11, 16, 17). Based on the definition applied, the incidence of second primaries varies significantly from 2.4 to 17% (11). An international consensus needs to be reached so that we can make obtaining comparable, clinically valuable information a priority.

By applying SEER’s criteria in a cohort of 10,857 CMM survivors, Bradford et al. reported a significantly increased risk for non-melanomatous malignancies, particularly for breast, prostate, and non-Hodgkin’s lymphoma (observed to expected ratio [E:O] = 1.10, 1.15, and 1.25, respectively) (18).

In this context, the sex-related incidence of multiple primaries is a critical issue that needs to be investigated further. A US study (on 117,000 CMM patients followed from 1992 to 2006) showed a sex-independent increase of subsequent thyroid cancer, NHL, and chronic lymphocytic leukemia, with a higher risk of second kidney and prostate cancers in males, and a higher risk of breast cancer in females (4).

To delve deeper into the effect of sex on cancer incidence, the current retrospective population-based study assessed data on multiple (synchronous and metachronous) primary malignancies in 9,726 CMM patients stratified by sex and followed up for 65,046 person-years.

### Synchronous and metachronous multiple cancers: the clinical impact of the definition

Our study shows that CMM patients carry a greater risk of multiple malignancies within 6 months of the index case. When patients develop a secondary primary cancer, these are categorized as either synchronous or metachronous primaries, depending on the length of time between the two cancers. However, the timeframe considered differs significantly according to SEER and IARC definitions. The SEER database considers any malignancy diagnosed within 2 months from the index cancer as synchronous, while the IARC considers any malignancy diagnosed within 6 months of the index cancer. This enormous nosology inconsistency results in significant differences in the epidemiological profile and the clinical interpretation of multiple primaries.

From an epidemiological viewpoint, inconsistent definitions result in unreliable data interpretation and comparisons. From a clinical viewpoint, real-world experience shows that the two-month interval is too narrow to accomplish all diagnostic/staging cancer procedures, thus increasing the relative number of metachronous malignancies recorded. By expanding the timeframe of synchronous malignancies to 6 months, the IARC definition is more consistent with the natural cancers’ history, and also supports the clinical-biological rationale of secondary prevention strategies. Thus, the present study considered all multiple malignancies in different sites diagnosed within 6 months from the index CMM as synchronous.

### Synchronous cancers

Consistent with the IARC conventional definition, synchronous cancers may plausibly be considered as side-effects of the diagnostic procedures triggered by the index cancer.

This study documented that the overall risk of synchronous cancers was higher in both sexes, irrespective of the site. Both sexes featured an excess risk for synchronous kidney/urinary malignancies, while females showed a short-term increased “detection” of breast cancer.

Although shared pathogenic risk factors cannot be excluded, the higher incidence of synchronous cancers could also be interpreted as an effect of increased access to the diagnostic imaging procedures that CMM patients undergo during the staging process of the index melanoma. These procedures may lead to the incidental discovery of asymptomatic/undiagnosed tumors in the first month after the index CMM (5, 19–22).

### Metachronous cancers in males

Among CMM male survivors, the present findings did not feature any overall excess of metachronous cancer risk. Consistent with previous studies however, male patients did feature an excess risk of metachronous prostate and thyroid malignancies (5). This increased thyroid cancer risk remained higher until 60 months after the index CMM, which could suggest the involvement of cancer-promoting genetic abnormalities (i.e., BRAFv600e, CDKN2A) which could potentially be involved in the pathogenesis of both malignancies (23–26). Due to the small number of tested cases (26), the real pathogenetic impact of these association(s) needs to be further confirmed. Beyond any cancer-prone molecular profile, the significant relationship between CMM and metachronous thyroid malignancies could also result from the increasing incidence of thyroid cancers (papillary and undifferentiated), the detection of which has increased due to more widespread access to diagnostic imaging procedures.

### Metachronous cancers in females

If we exclude skin melanomatous and non-melanomatous malignancies, females consistently featured an overall excess of metachronous cancer risk. When looking at single cancer sites, breast, kidney/urinary tract, and hematological (NHL) malignancies also showed a significant excess of risk. This is in line with a previously-reported two-way association between invasive CMM and NHL (27–29). The present findings thus further support the hypothesis that the two malignancies may share similar risk factors (4), such as impaired immunological status (30–32) or genetic susceptibility (i.e., chromosome 9p21 deletion) (33–36).

While the increased risk of synchronous breast cancer likely results from the increased medical surveillance triggered by the CMM staging protocols, the excess of breast cancer risk (which can be confirmed up until 10 years after the index-CMM) also suggests a potential pathogenetic involvement of BRCA2 and CDKN2A mutations (37–40). Thus, performing next-generation sequencing (NGS) analysis could be useful not only in detecting tumor heterogeneity and potentially finding new targetable mutations for systemic drug therapies, but also in verifying potential genetic profiles that could predict metachronous and synchronous tumor risk (41).

### Strengths and limitations

An important limitation of the present study is the lack of detailed information on environmental cancer risk factors, including socioeconomic status, genetic variants, and behavioral characteristics: collecting this level of patient-profiling data will be the next frontier of high-resolution cancer registration. Furthermore, only a fraction of the regional population was covered by the cancer registry in the first few years assessed in this study, which could affect generalizability. Nonetheless, the historical registration area yielded incidence estimates for various cancer sites that were proven to be comparable with those available for the whole population area.

## Conclusion

The present study found a significantly increased risk of synchronous and metachronous cancers in survivors of CMM, especially in terms of prostate, thyroid, or kidney and urinary tract cancers in men, and NHL, breast, or kidney and urinary tract cancers in women. Studies on multiple primary cancers may provide powerful insights into cancer etiology, including the cancer-promoting role of environmental and genetic risk factors.

In CMM cancer patients, the results of this study provide the clinical rationale for developing secondary prevention strategies (including counseling), as well as decision-supporting tools. Moreover, in CMM survivors, the present results add clinically helpful information for sex-tailored surveillance protocols. Patients diagnosed with melanoma should therefore remain under surveillance, not only for recurrences but also for new primary melanomas and other cancers.

## Data availability statement

The raw data supporting the conclusions of this article will be made available by the authors, without undue reservation.

## Ethics statement

The studies involving human participants were reviewed and approved by Veneto Oncological Institute’s Ethics Committee (No. 52/2016). Written informed consent for participation was not required for this study in accordance with the national legislation and the institutional requirements.

## Author contributions

AB, SG, MR, and SM: conceptualization. PF and MZ: methodology, software, and formal analysis. AB, EB, ST, LD, and CR: investigation. MZ: data curation. PF, ST, LD, CR, and SM: visualization. GG and MM: writing – original draft preparation. AB, SM, and MR: writing – editing supervision. All authors contributed to the article and approved the submitted version.

## Funding

This research has received “Current Research” funds from the Italian Ministry of Health to cover publication costs.

## Conflict of interest

The authors declare that the research was conducted in the absence of any commercial or financial relationships that could be construed as a potential conflict of interest.

## Publisher’s note

All claims expressed in this article are solely those of the authors and do not necessarily represent those of their affiliated organizations, or those of the publisher, the editors and the reviewers. Any product that may be evaluated in this article, or claim that may be made by its manufacturer, is not guaranteed or endorsed by the publisher.

## References

[1] Ahmed B Qadir MI Ghafoor S. Malignant melanoma: skin cancer-diagnosis, prevention, and treatment. Crit Rev Eukaryot Gene Expr. (2020) 30:291–7. doi: 10.1615/CritRevEukaryotGeneExpr.2020028454, PMID: 32894659

[2] Saginala K Barsouk A Aluru JS Rawla P Barsouk A. Epidemiology of melanoma. Med Sci (Basel). (2021) 9:63. doi: 10.3390/medsci9040063 34698235 PMC8544364

[3] Associazione Italiana di Oncologia Medica, Gruppo di Lavoro Registri Tumori Italiani, SIAPEC-IAP. I Numeri del Cancro in Italia; (2021). Available at: https://www.aiom.it/wp-content/uploads/2021/10/2021_NumeriCancro_web.pdf. (Accessed November 15, 2022).

[4] Balamurugan A Rees JR Kosary C Rim SH Li J Stewart SL. Subsequent primary cancers among men and women with in situ and invasive melanoma of the skin. J Am Acad Dermatol. (2011) 65:S69–77. doi: 10.1016/j.jaad.2011.04.033, PMID: 22018070

[5] Caini S Boniol M Botteri E Tosti G Bazolli B Russell-Edu W. The risk of developing a second primary cancer in melanoma patients: a comprehensive review of the literature and meta-analysis. J Dermatol Sci. (2014) 75:3–9. doi: 10.1016/j.jdermsci.2014.02.007, PMID: 24680127

[6] Vogt A Schmid S Heinimann K Frick H Herrmann C Cerny T. Multiple primary tumours: challenges and approaches, a review. ESMO Open. (2017) 2:e000172. doi: 10.1136/esmoopen-2017-000172, PMID: 28761745 PMC5519797

[7] ISTAT. Popolazione al 31 Dicembre – Totale Regione: Veneto Periodo: Anno; (2022). Available at: https://demo.istat.it/app/?i=CDQ&l=it. (Assessed May 11, 2023)

[8] ISTAT. Tavole di Mortalità della Popolazione Residente - Serie Storica per Ripartizione/Regione/Provincia; (2022). Available at: http://demo.istat.it/tvm2016/index.php?lingua=ita (Accessed December 13, 2022).

[9] Toniolo F Mantoan D Maresso A. Veneto Region, Italy. Health system review. Health Syst Transit. (2012) 14:i–xix, 1–138. 22575766

[10] Ferretti S. Airtum Cancer Registration Handbook. Italy: Florence (2009).

[11] Copur MS Manapuram S. Multiple primary tumors over a lifetime. Oncol Williston Park N. (2019) 33:629384 31365752

[12] Curtis RE Boice JD Kleinerman RA Flannery JT Fraumeni JF. Summary: multiple primary cancers in Connecticut, 1935-82. Natl Cancer Inst Monogr. (1985) 68:219–42. PMID: 4088299

[13] Breslow NE Day NE. Statistical methods in cancer research. Volume II--the design and analysis of cohort studies. IARC Sci Publ. (1987) 82:1–406. 3329634

[14] Benjamini Y Hochberg Y. Controlling the false discovery rate: a practical and powerful approach to multiple testing. J R Stat Soc Series B Stat Methodol. (1995) 57:289–300. doi: 10.1111/j.2517-6161.1995.tb02031.x

[15] SEER*Stat Software. (2023). Available at: https://seer.cancer.gov/seerstat/ (Accessed March 10, 2023)

[16] Herrmann C Cerny T Savidan A Vounatsou P Konzelmann I Bouchardy C. Cancer survivors in Switzerland: a rapidly growing population to care for. BMC Cancer. (2013) 13:287. doi: 10.1186/1471-2407-13-287, PMID: 23764068 PMC3685597

[17] Wood ME Vogel V Ng A Foxhall L Goodwin P Travis LB. Second malignant neoplasms: assessment and strategies for risk reduction. J Clin Oncol Off J Am Soc Clin Oncol. (2012) 30:3734–45. doi: 10.1200/JCO.2012.41.8681 23008293

[18] Bradford PT Freedman DM Goldstein AM Tucker MA. Increased risk of second primary cancers after a diagnosis of melanoma. Arch Dermatol. (2010) 146:265–72. doi: 10.1001/archdermatol.2010.2, PMID: 20231496 PMC3076705

[19] Crocetti E Guzzinati S Paci E Falcini F Zanetti R Vercelli M. The risk of developing a second, different, cancer among 14 560 survivors of malignant cutaneous melanoma: a study by AIRTUM (the Italian network of Cancer registries). Melanoma Res. (2008) 18:230–4. doi: 10.1097/CMR.0b013e3282fafd0a, PMID: 18477899

[20] Schmid-Wendtner MH Baumert J Wendtner CM Plewig G Volkenandt M. Risk of second primary malignancies in patients with cutaneous melanoma. Br J Dermatol. (2001) 145:981–5. doi: 10.1046/j.1365-2133.2001.04507.x 11899153

[21] Wu YH Kim GH Wagner JD Hood AF Chuang TY. The association between malignant melanoma and noncutaneous malignancies. Int J Dermatol. (2006) 45:529–34. doi: 10.1111/j.1365-4632.2005.02640.x 16700785

[22] Curtis RW Ries AG. Methods In: Curtis RE Freedman DM Ron E LAG R Hacker DG Edwards BK, editors. New Malignancies among Cancer Survivors: SEER Cancer Registries, 1973–2000. Bethesda, MD: National Cancer Institute. NIH Publ. No. 05–5302 (2006). 9–14.

[23] Fargnoli MC Argenziano G Zalaudek I Peris K. High- and low-penetrance cutaneous melanoma susceptibility genes. Expert Rev Anticancer Ther. (2006) 6:657–70. doi: 10.1586/14737140.6.5.657, PMID: 16759158

[24] Yang GB Barnholtz-Sloan JS Chen Y Bordeaux JS. Risk and survival of cutaneous melanoma diagnosed subsequent to a previous cancer. Arch Dermatol. (2011) 147:1395–402. doi: 10.1001/archdermatol.2011.1133, PMID: 22184761

[25] Stenman A Yang M Paulsson JO Zedenius J Paulsson K Juhlin CC. Pan-genomic sequencing reveals actionable CDKN2A/2B deletions and Kataegis in anaplastic thyroid carcinoma. Cancers. (2021) 13:6340. doi: 10.3390/cancers13246340, PMID: 34944959 PMC8699293

[26] Oakley GM Curtin K Layfield L Jarboe E Buchmann LO Hunt JP. Increased melanoma risk in individuals with papillary thyroid carcinoma. JAMA Otolaryngol Head Neck Surg. (2014) 140:423–7. doi: 10.1001/jamaoto.2014.78 24626334

[27] Brennan P Scélo G Hemminki K Mellemkjaer L Tracey E Andersen A. Second primary cancers among 109 000 cases of non-Hodgkin’s lymphoma. Br J Cancer. (2005) 93:159–66. doi: 10.1038/sj.bjc.6602654, PMID: 15970927 PMC2361473

[28] Travis LB Curtis RE Boice JD Hankey BF Fraumeni JF. Second cancers following non-Hodgkin’s lymphoma. Cancer. (1991) 67:2002–9. doi: 10.1002/1097-0142(19910401)67:7<2002::AID-CNCR2820670729>3.0.CO;2-E 2004317

[29] Goggins WB Finkelstein DM Tsao H. Evidence for an association between cutaneous melanoma and non-Hodgkin lymphoma. Cancer. (2001) 91:874–80. doi: 10.1002/1097-0142(20010215)91:4<874::AID-CNCR1076>3.0.CO;2-O 11241258

[30] Greene MH Young TI Clark WH. Malignant melanoma in renal-transplant recipients. Lancet Lond Engl. (1981) 317:1196–9. doi: 10.1016/S0140-6736(81)92359-X 6112537

[31] Curtis RE Rowlings PA Deeg HJ Shriner DA Socíe G Travis LB. Solid cancers after bone marrow transplantation. N Engl J Med. (1997) 336:897–904. doi: 10.1056/NEJM199703273361301 9070469

[32] Shiels MS Engels EA. Evolving epidemiology of HIV-associated malignancies. Curr Opin HIV AIDS. (2017) 12:6–11. doi: 10.1097/COH.0000000000000327, PMID: 27749369 PMC5240042

[33] Frigerio S Disciglio V Manoukian S Peissel B Della Torre G Maurichi A. A large de novo 9p21.3 deletion in a girl affected by astrocytoma and multiple melanoma. BMC Med Genet. (2014) 15:59. doi: 10.1186/1471-2350-15-59, PMID: 24884915 PMC4036080

[34] Guney S Bertrand P Jardin F Ruminy P Kerckaert JP Tilly H. Molecular characterization of 9p21 deletions shows a minimal common deleted region removing CDKN2A exon 1 and CDKN2B exon 2 in diffuse large B-cell lymphomas. Genes Chromosomes Cancer. (2011) 50:715–25. doi: 10.1002/gcc.20893, PMID: 21638516

[35] Pollak C Hagemeijer A. Abnormalities of the short arm of chromosome 9 with partial loss of material in hematological disorders. Leukemia. (1987) 1:541–8. PMID: 3312844

[36] Fountain JW Karayiorgou M Ernstoff MS Kirkwood JM Vlock DR Titus-Ernstoff L. Homozygous deletions within human chromosome band 9p21 in melanoma. Proc Natl Acad Sci U S A. (1992) 89:10557–61. doi: 10.1073/pnas.89.21.10557, PMID: 1438246 PMC50378

[37] Goggins W Gao W Tsao H. Association between female breast cancer and cutaneous melanoma. Int J Cancer. (2004) 111:792–4. doi: 10.1002/ijc.20322 15252852

[38] Breast Cancer Linkage Consortium. Cancer risks in BRCA2 mutation carriers. J Natl Cancer Inst. (1999) 91:1310–6. doi: 10.1093/jnci/91.15.1310 10433620

[39] Borg A Sandberg T Nilsson K Johannsson O Klinker M Måsbäck A. High frequency of multiple melanomas and breast and pancreas carcinomas in CDKN2A mutation-positive melanoma families. J Natl Cancer Inst. (2000) 92:1260–6. doi: 10.1093/jnci/92.15.1260, PMID: 10922411

[40] Tucker MA Goldstein AM. Melanoma etiology: where are we? Oncogene. (2003) 22:3042–52. doi: 10.1038/sj.onc.1206444 12789279

[41] Diefenbach RJ Lee JH Menzies AM Carlino MS Long GV Saw RPM. Design and testing of a custom melanoma next generation sequencing panel for analysis of circulating tumor DNA. Cancers (Basel). (2020) 12:2228. doi: 10.3390/cancers12082228. PMID: 32785074 PMC7465941

