# Supplementary material for: Synchronous and metachronous multiple primary cancers in melanoma survivors: a gender perspective
Source: Front Public Health. 2023 Jun 16;11:1195458. doi: 10.3389/fpubh.2023.1195458 (PMC10313207; doi:10.3389/fpubh.2023.1195458)
Supplement: Supplementary file 1 [file Data_Sheet_1.docx]

**Supplementary Table 1.** Average population covered by the Veneto Cancer Registry (RTV).

| Years | Average population covered by cancer registry | % coverage |
| --- | --- | --- |
| 1987 | 1,154,307 | 27% |
| 1988-1989 | 1,455,439 | 33% |
| 1990-1997 | 1,970,830 | 45% |
| 1998-2007 | 2,239,770 | 49% |
| 2008-2012 | 2,577,652 | 53% |
| 2013 | 4,687,492 | 96% |
| 2014-2019 | 4,892,209 | 100% |

**Supplementary Table 2.** Age-standardized incidence rates for the historical area (HA) and whole region for the main cancer sites. Period 2014-2019.

|  | Men | | | | Women | | | |
| --- | --- | --- | --- | --- | --- | --- | --- | --- |
|  | HA | Region | Rate ratio | p-value | HA | Region | Rate ratio | p-value |
| All malignant tumors *excluding in-situ* cutaneous melanoma and non-melanoma skin cancer | 690.0 | 690.7 | 1.001 | 0.8585 | 516.5 | 512.8 | 0.9929 | 0.2377 |
| Breast |  |  |  |  | 173.1 | 169.9 | 0.9816 | 0.0796 |
| Prostate | 148.3 | 147.4 | 0.9942 | 0.6407 |  |  |  |  |
| Colon, rectum, and anus | 79.5 | 79.9 | 1.004 | 0.8182 | 50.8 | 50.1 | 0.986 | 0.456 |
| Urinary Bladder | 66.4 | 66.5 | 1.0021 | 0.9203 | 15.1 | 14.0 | 0.931 | 0.0433 |
| Lung, bronchus, and trachea | 83.0 | 84.0 | 1.0117 | 0.489 | 34.3 | 33.6 | 0.9798 | 0.3825 |
| Invasive cutaneous melanoma | 33.4 | 31.9 | 0.9564 | 0.0886 | 25.8 | 24.6 | 0.9535 | 0.0963 |

The rate ratios between the standardized rates in the historical area and the whole region showed no significant differences. For all malignant tumors, the standardized rate in the historical area was 690.0 vs. 690.7 for the whole region in men, and 516.5 vs. 512.8 in women, with rate ratios of 1.001 and 0.9929, respectively (both p-values are higher than 0.05).
